# Long‐term outcomes of arthroscopic Bankart repair with additional posteroinferior capsular plication in patients with anterior shoulder instability and hyperlaxity: Minimum 10‐year follow‐up

**DOI:** 10.1002/jeo2.70735

**Published:** 2026-05-04

**Authors:** Anh Do, Agahan Hayta, Philipp Moroder, Markus Scheibel, Rony‐Orijit Dey Hazra, Alp Paksoy, Doruk Akgün

**Affiliations:** ^1^ Center for Musculoskeletal Surgery Charité University Hospital Berlin Germany; ^2^ Schulthess Klinik, Department of Shoulder and Elbow Surgery Zurich Switzerland

**Keywords:** arthroscopy, bankart repair, capsular plication, hyperlaxity, shoulder instability

## Abstract

**Purpose:**

To evaluate the long‐term outcomes of arthroscopic Bankart repair with additional posteroinferior capsular plication in patients with anterior shoulder instability and hyperlaxity, and to compare the outcomes of posteroinferior capsular plication using suture‐only fixation versus suture anchor fixation.

**Methods:**

In this retrospective study, patients were included who underwent arthroscopic Bankart repair and additional posteroinferior capsular plication for anterior shoulder instability and hyperlaxity (type B3) between 2006 and 2014 at our institution. Primary outcome was recurrent instability. Secondary outcomes were Subjective Shoulder Value (SSV), visual analog scale (VAS), Constant score (CS), Western Ontario Shoulder Instability Index (WOSI) and Rowe score, as well as return to sport.

**Results:**

Of 54 included shoulders, 33 shoulders in 32 patients (61.1%) were evaluated after a mean follow‐up of 13.2 ± 2.3 years. The overall recurrence rate was 18.2% (6/33). The total revision rate was 9.1%, with two revisions due to recurrent instability and one due to posteroinferior knot impingement. The number of preoperative dislocations correlated negatively with the CS (*ρ* = −0.425, *p* = 0.019) and the WOSI (*ρ* = −0.471, *p* = 0.009). A total of 97% of all patients returned to sports, with 57.6% returning to 90%–100% of their preoperative sports activity. Posteroinferior capsular plication using suture‐only fixation was associated with a higher recurrence rate (3/6, 50%), compared to the use of suture anchor fixation (3/27, 11.1%; *p* = 0.025).

**Conclusion:**

Arthroscopic Bankart repair combined with posteroinferior capsular plication provided durable long‐term shoulder function and reliable return to sport in patients with anterior instability and hyperlaxity. Performing the posteroinferior plication with suture anchor fixation might be associated with lower recurrence rates compared to suture‐only plication. Clinical outcomes declined with an increasing number of preoperative dislocations.

**Level of Evidence:**

Level III, cohort study.

**Abbreviations**
CS: Constant score
IQR: interquartile range
No: number
SSV: Subjective Shoulder Value
VAS: visual analog scale
WOSI: Western Ontario Shoulder Instability Index

## INTRODUCTION

Hyperlaxity has been identified as a common risk factor for recurrent instability after arthroscopic Bankart repair, affecting 5%–15% of patients with shoulder instability [1, 6, 23, 30]. In contrast to patients with unidirectional instability without hyperlaxity (type B2 according to Gerber) [13], patients with hyperlaxity (type B3) typically experience subluxations rather than dislocations, and concomitant structural lesions, such as Bankart lesions or Hill–Sachs defects, are less common [12, 17, 30]. If structural lesions are present or if nonoperative treatment fails, the optimal surgical procedure remains controversial. The goal of the surgery is to restore the anatomy and to reduce the excessive capsular volume by retensioning the static capsulolabral stabilisers [17, 30]. The literature suggests that isolated Bankart repair may be insufficient for patients with hyperlaxity and has been associated with recurrence rates of up to 60% in high‐risk populations [27]. Surgical options for patients with hyperlaxity include rotator interval closure, capsular shift or plication [35], arthroscopic subscapularis augmentation [20] and Trillat procedure [5].

Arthroscopic Bankart repair combined with posteroinferior capsular plication is considered the gold standard for patients with anterior shoulder instability and hyperlaxity without critical bone loss [29] and has demonstrated favourable short‐term results [34]. Biomechanical studies have shown restoration of the physiological capsular volume in patients with anterior shoulder instability [10, 31]. However, long‐term outcomes of this procedure in patients with hyperlaxity have not yet been reported. Two different techniques of posteroinferior capsular plication have been described: suture‐only fixation and suture anchor fixation. To date, no study has compared the effectiveness and outcomes of these two techniques.

The purpose of the present study was to evaluate the long‐term clinical outcomes after arthroscopic Bankart repair with additional posteroinferior capsular plication in patients with anterior shoulder instability and hyperlaxity without significant bone loss. The outcomes of posteroinferior capsular plication using suture‐only fixation were compared to those using suture anchor fixation. Furthermore, risk factors for recurrent instability were analysed. We hypothesised that arthroscopic Bankart repair combined with additional posteroinferior capsular plication would result in good clinical outcomes and an acceptable recurrence rate in patients with anterior shoulder instability and hyperlaxity at a minimum follow‐up of 10 years.

## METHODS

### Study cohort

In this retrospective study, patients were included who underwent arthroscopic Bankart repair and additional posteroinferior capsular plication for anterior shoulder instability and hyperlaxity (type B3) between 2006 and 2014 at our institution. The type of instability was classified preoperatively according to Gerber and Nyffeler [13], using the hyperabduction test described by Gagey and Gagey [11], and was documented in the operative reports. Patients with a positive Gagey test were classified as type B3, indicating local shoulder hyperlaxity. Exclusion criteria included multidirectional instability, previous shoulder surgeries, concomitant shoulder injuries requiring surgery, such as Superior Labrum Anterior and Posterior lesions or rotator cuff tears, the use of fewer than three anchors for anterior Bankart repair, and indication for bony reconstruction (critical bony defects: glenoid bone defect >20% of the surface area, off‐track Hill‐Sachs lesion). No patients in the cohort underwent additional stabilisation methods, such as remplissage, or reoperation of the same shoulder unrelated to the instability problem.

### Surgical technique and postoperative management

The surgeries were performed with the patient under general anaesthesia in the lateral decubitus position. Three standard portals were used: posterior, anterosuperior and anteroinferior. A diagnostic arthroscopy was conducted through the standard posterior portal to assess concomitant injuries and revealed an anteroinferior capsulolabral lesion as well as excessive capsular volume in all patients.

First, the Bankart repair was performed. For this procedure, three different anchor designs were used sequentially to reattach the labrum: Bio‐FASTak (*n* = 3, Arthrex, hard‐body anchor), PushLock (*n* = 26, Arthrex, hard‐body anchor), Lupine (*n* = 1, Depuy Mitek, hard‐body anchor) and JuggerKnot (*n* = 3, Zimmer Biomet, all‐suture anchor). For the Bio‐FASTak, Lupine and JuggerKnot anchors the anchor‐first technique was used, whereas for the PushLock anchor, the knotless suture‐first technique was applied. The capsulolabral complex was mobilised, and the glenoid rim was freshened.

For the anchor‐first technique, the first anchor was placed at the 5:30 o'clock position (hourglass position in a right shoulder) at the anterior glenoid rim. Using a suture lasso, both sutures were passed through the anterior capsulolabral complex and tied to form a mattress stitch that reattached and shifted the complex. At least three anchors were used, with these steps being repeated accordingly.

For the suture‐first technique, a FiberWire suture (Arthrex) was shuttled through the capsulolabral complex using a suture lasso to form a cinch stitch. The suture was then inserted into the anchor, which was subsequently placed into a predrilled hole. These steps were repeated with at least three anchors to reattach and shift the capsulolabral complex.

Afterwards, the posteroinferior capsular plication was performed using either a suture anchor fixation (after 2010) or a suture‐only fixation (before 2010). Using a suture anchor fixation, the capsular plication was performed similarly to the technique described above, with an additional anchor inserted at the 7 o'clock position (in the right shoulder; 5 o'clock in the left shoulder) to plicate the posteroinferior capsule. For the plication, three different anchors were used: the PushLock (*n* = 22), the Lupine (*n* = 1) and the JuggerKnot (*n* = 4).

**Figure 1:**
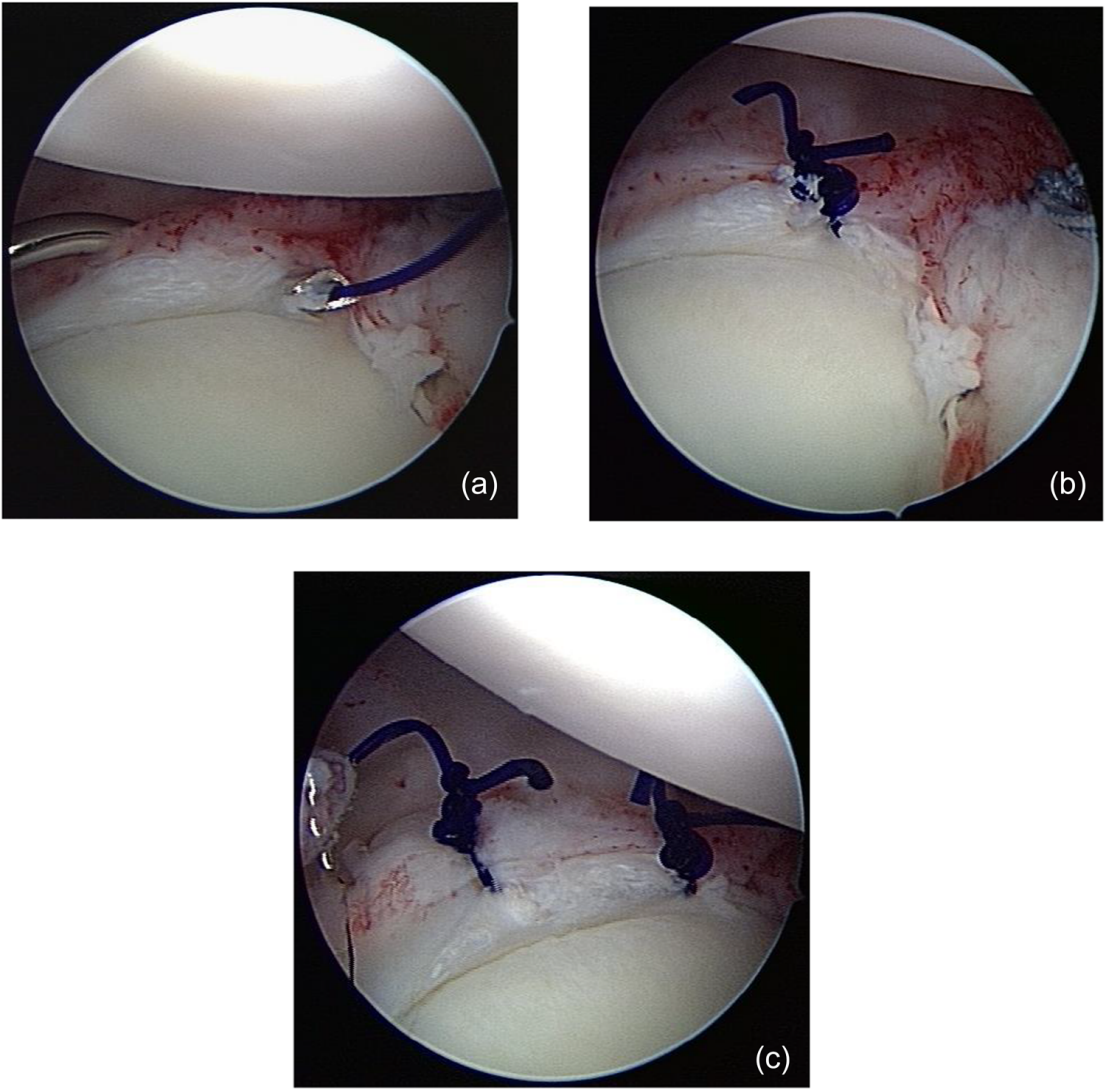
Intraoperative arthroscopic images of suture‐only posteroinferior capsular plication with PDS suture in the left shoulder. (a) Using a suture lasso, a PDS suture is passed through the capsule approximately 1 cm from the labrum, reintroduced into the joint, and subsequently passed through the labrum at the 7 o'clock position. (b) The suture is tied using a sliding knot to achieve capsular plication. (c) Arthroscopic view demonstrating two posteroinferior capsular plications at the 7 and 8 o'clock positions.

The posteroinferior capsular plication using a suture‐only fixation was performed as described by Wolf et al. [36] (Figure 1). Using a suture lasso, a polydioxanone (PDS) suture (Ethicon) was passed down through the capsule approximately 1 cm from the labrum, up through the capsule re‐entering the joint, then through the labrum at the 7 o'clock position (Figure 1a), and knotted with a sliding knot to plicate it onto itself (Figure 1b). These steps were repeated with a second PDS suture at the 8 o'clock position to reduce the capsular volume (Figure 1c).

After surgery, all patients followed a standardised rehabilitation protocol for arthroscopic shoulder stabilisation at our department. The shoulder was immobilised in a neutral position in a shoulder sling for 4 weeks. During this period, passive mobilisation exercises were permitted up to 60° of flexion and abduction, and 0° of external rotation. After 4 weeks, active‐assisted exercises were initiated, limited to 90° of flexion and abduction, and 0° of external rotation. Active range of motion without restriction and strengthening exercises for the rotator cuff and deltoid were allowed after 6 weeks, with sport‐specific training beginning after 12 weeks.

### Radiological evaluation

To assess the preoperative glenoid defects in percentage, the PICO method was used in the en face view of computed tomography (CT) scans [2, 3]. The Hill–Sachs lesions were evaluated in magnetic resonance imaging (MRI) using the on‐track off‐track method described by Di Giacomo et al. [8, 15]. All patients had undergone preoperative imaging. However, in nine shoulders, the original CT or MRI images were unavailable due to limitations of the storage system. In these cases, intraoperative arthroscopic images and operative reports were used to assess the glenoid defect and Hill–Sachs lesion. Critical glenoid defects (>20%) and off‐track Hill–Sachs lesions were considered indications for bony reconstruction, and therefore, these patients were excluded from the study.

### Clinical evaluation

The primary outcome of this study was recurrent instability, defined as dislocation or subluxation. The secondary outcomes were shoulder function and return to sports. Patients were invited to a follow‐up examination at our institution and were evaluated by an independent examiner with a medical history of shoulder instability, a standard clinical assessment of both shoulders, and questionnaires assessing shoulder outcome scores. To assess the history of shoulder instability, the number, dates and causes of subluxations and dislocations before and after surgery, as well as revision surgeries, and return to work were documented. All types of sports, the sports activity level according to Valderrabano et al. [32] preoperatively, postoperatively (highest level after surgery) and at present, and the recovery of athletic activity according to Rhee et al. [26] were recorded. The physical examination included the assessment of range of motion, shoulder hyperlaxity using the Gagey test [11] on the contralateral side, generalised hyperlaxity using the Beighton score [4], anterior apprehension and scapular dyskinesis classified according to Kibler et al. [18]. The apprehension test was regarded as positive if the patient described a sense of instability.

To assess the shoulder function, the following shoulder outcome scores were used: Subjective Shoulder Value (SSV) [14], visual analog scale (VAS) for pain level at rest and during movement, Constant score (CS) [7], Western Ontario Shoulder Instability Index (WOSI) [19] and Rowe score [28]. The WOSI was converted to a 0%–100% scale, with a higher percentage indicating a better shoulder function. Patients who underwent revision surgery were not included in the secondary outcomes.

Subgroup analyses comparing patients with recurrence and those without recurrence were performed to assess potential risk factors for recurrence, including sex, age at surgery, hand dominance, level of sports activity, hyperlaxity, bony defects, number of preoperative dislocations and time from first dislocation to surgery.

### Statistical analysis

Statistical analysis was performed using IBM SPSS Statistics software (Version 29; IBM), with a *p*‐value of <0.05 considered statistically significant. The normal distribution of the data was tested using the Kolmogorov–Smirnov test. Categorical variables were reported as frequencies and percentages, whereas continuous variables were presented as mean ± standard deviation (for normally distributed data) or as median (Q1–Q3 interquartile range) (for non‐normally distributed data). Continuous variables between two groups were compared using the independent *t*‐test (for normally distributed data) and the Mann–Whitney *U*‐test (for non‐normally distributed data). To compare continuous variables between preoperatively and postoperatively, the paired *t‐*test was performed. The chi‐square test was used for dichotomous data. To assess the correlation between continuous variables and outcome measures, the Spearman correlation coefficient was calculated. Kaplan–Meier survival analysis was conducted to evaluate the time from surgery to recurrence, with the log‐rank test employed to compare survival times. Cox regression analysis was used to assess hazard ratios. To analyse the influence of potential risk factors on recurrent instability and to calculate the odds ratios with 95% confidence intervals, binary logistic regression was performed.

## RESULTS

**Figure 2:**
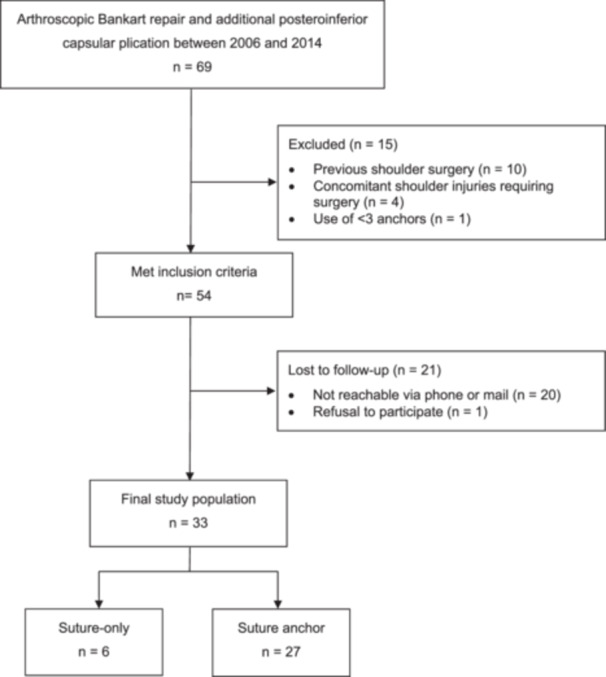
Flowchart of patient enrolment.

Of 69 shoulders, 54 met the eligibility criteria and were invited for an in‐person evaluation after a minimum follow‐up of 10 years (flowchart of study enrolment in Figure 2). Of these, 21 were either not reachable or declined participation. A total of 33 shoulders in 32 patients (61.1%) were evaluated after a mean follow‐up of 13.2 ± 2.3 years.

The patient characteristics are presented in Table 1.

**Table 1:** Patient characteristics.

Variable	All patients *n* = 33 (100%)	Suture‐only plication *n* = 6 (18.2%)	Suture anchor plication *n* = 27 (81.8%)	*p*
Follow‐up period, mean ± SD, years	13.2 ± 2.3	16.4 ± 1.6	12.5 ± 1.7	**<0.001**
Male sex, *n* (%)	20 (60.6)	3 (50)	17 (63)	0.557
Dominant arm affected, *n* (%)	16 (48.5)	4 (66.7)	12 (44.4)	0.325
Beighton score, *n* (%)				0.252
<4	28 (84.8)	6 (100)	22 (81.5)	
≥4	5 (15.2)	0 (0)	5 (18.5)	
Mechanism of first dislocation, *n* (%)				0.475
Traumatic	30 (90.9)	5 (83.3)	25 (92.6)	
Atraumatic	3 (9.1)	1 (16.7)	2 (7.4)	
No. of preoperative dislocations, median (IQR)	3 (2–8)	2 (1–11.3)	3 (2–8)	0.227
1, *n* (%)	3 (9.1)	2 (33.3)	1 (3.7)	
>1, *n* (%)	30 (90.9)	4 (66.6)	26 (96.3)	
Age at surgery, mean ± SD, years	28.2 ± 9.3	23.3 ± 9.7	29.3 ± 9	0.078
Time to surgery, median (IQR), months	37.9 (14.6–116.7)	20.9 (1.2–30.6)	60.4 (16.3–127.8)	**0.040**
Glenoid defect in %, median (IQR)^a^	3.5 (0–6)	0 (0–0)	3.6 (0–7.1)	0.165
Type of suture anchors Bankart repair, *n* (%)				0.315
All‐suture	4 (12.1)	0 (0)	4 (14.8)	
Hard‐body	29 (87.9)	6 (100)	23 (85.2)	
No. of anchors Bankart repair, median (IQR)	3 (3–3)	3 (3–3)	3 (3–3)	0.515

*Note*: Bold values indicate statistically significant at *p* < 0.05.

Abbreviations: IQR, interquartile range; No., number; SD, standard deviation.

^a^ Data are available for 24 patients.

### Recurrent instability

Recurrent instability was observed in 18.2% (6/33) of shoulders at follow‐up, comprising 15.2% (5/33) redislocations and 3% (1/33) subluxations. Half of the recurrences (3/6) occurred after a new traumatic event, and the other half occurred during daily activities. The median number of recurrences was 2 (1–7.25) until last follow‐up.

**Figure 3:**
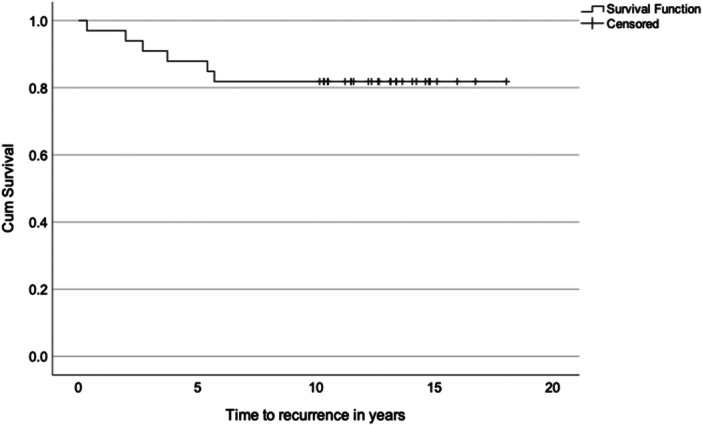
Kaplan–Meier survival curve of recurrence rate.

The mean time from surgery to recurrence was 3.3 ± 2.1 years. Recurrent instability developed within the first 2 years after surgery in two patients (33.3%; one in the suture‐only fixation group and one in the suture anchor fixation group) and between 2 and 6 years after surgery in the remaining four patients (66.6%; two in the suture‐only fixation group and two in the suture anchor fixation group) (Figure 3).

The overall revision rate was 9.1%, with two revisions due to recurrent instability and one due to posteroinferior knot impingement with a humeral chondral lesion. All revision cases underwent a second arthroscopic Bankart repair.

### Comparison between posteroinferior capsular plication techniques

**Figure 4:**
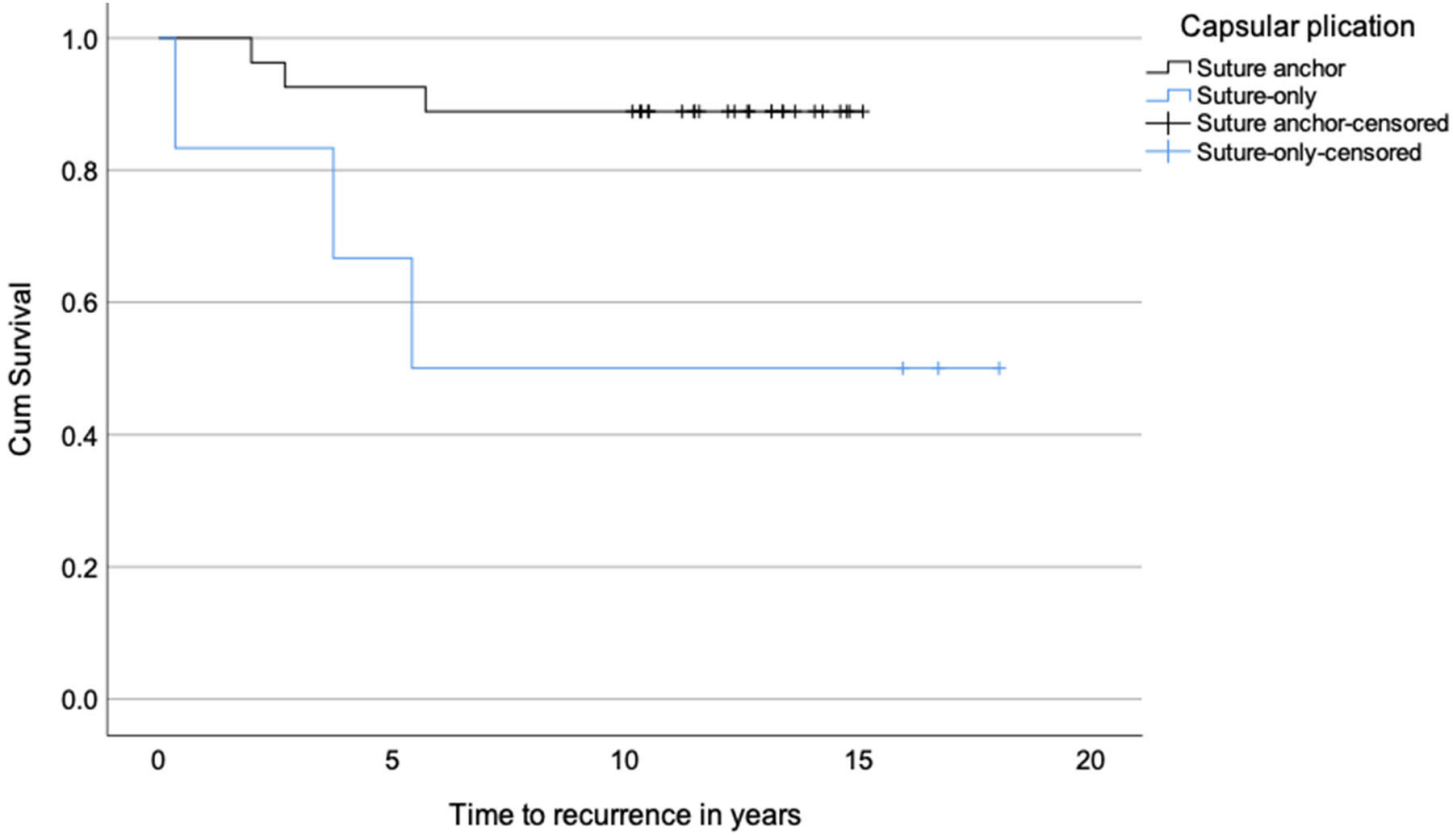
Kaplan–Meier survival curve of recurrence rate for posteroinferior capsular plication using suture‐only fixation and suture anchor fixation.

Posteroinferior capsular plication using suture‐only fixation was associated with a statistically significant higher recurrence rate (50%, 3/6), compared to suture anchor fixation (11.1%, 3/27; *p* = 0.025). The hazard ratio for recurrent instability in patients who underwent capsular plication with suture‐only fixation versus suture anchor fixation was 5.7 (*p* = 0.035, 95% confidence interval, 1.1–28.3) (Figure 4).

### Risk factors for recurrent instability

The recurrence rate was statistically significant higher in patients with an atraumatic first dislocation (66.7%, 2/3) compared to patients with a traumatic first dislocation (13.3%, 4/30; *p* = 0.022).

Sex (*p* = 0.131) and age (*p* = 0.712) did not affect the recurrence rate.

### Shoulder function

The shoulder outcome scores and clinical tests for patients without revision surgery (30 shoulders) are shown in Table 2. Patients without recurrent instability showed significant higher Rowe Scores compared to patients with recurrence (*p* ≤ 0.001). The outcome scores of the suture‐only group were comparable to those of the suture anchor group. Of the 26 shoulders without recurrent instability events, 5 shoulders (19.2%) demonstrated a positive apprehension test.

**Table 2:** Shoulder outcome scores and clinical tests of patients with and without recurrent instability.

Score	All patients (*n* = 33) median (IQR)	No recurrence (*n* = 26) median (IQR)	Recurrence (*n* = 4) median (IQR)	*p*
SSV	88.4% (80%–100%)	94% (80%–100%)	85% (65%–90%)	0.123
VAS for pain (rest)	0 (0–0)	0 (0–0)	0 (0–0)	0.930
VAS for pain (movement)	0 (0–1)	0 (0–1.25)	0 (0–0)	0.245
Constant score	87 (82.8–94)	88 (82.5–94.3)	85 (82.25–94.5)	0.659
WOSI	91.5% (81.1%–97.1%)	91.5% (82%–97.2%)	86% (58.6%–97.5%)	0.576
Rowe score	87.5 (75–100)	95 (75–100)	45 (33.8–45)	**<0.001**
Scapular dyskinesis, %	36	36.4	33.3	0.918
Apprehension, *n* (%)	6 (18.2)	5 (19.2%)	1 (25%)	0.788

*Note*: Bold *p*‐values are statistically significant (*p* < 0.05).

Abbreviations: IQR, interquartile range; SSV, subjective shoulder value; VAS, visual analog scale.

The number of preoperative dislocations was negatively correlated with the CS (*p* = 0.019) and the WOSI (*p* = 0.009) (Table 3). Additionally, a negative correlation was observed between the time from first dislocation to surgery and both the CS (*p* = 0.075) and WOSI (*p* = 0.076), although these were not statistically significant.

**Table 3:** Sports activity level according to Valderrabano preoperative, postoperative and at follow‐up.

	Preoperative	Postoperative	At Follow‐Up
Sports activity level	*n* (%)	*n* (%)	*n* (%)
0 (No sports activity)	2 (6.1%)	2 (6.1%)	6 (18.2%)
1 (Moderate, <1 h/week)	2 (6.1%)	1 (3%)	3 (9.1%)
2 (Normal, 1–5 h/week)	16 (48.5%)	19 (57.5%)	19 (57.5%)
3 (High, >5 h/week)	9 (27.3%)	9 (27.3%)	5 (15.2%)
4 (Competitive sport)	4 (12%)	2 (6.1%)	0 (0%)
Mean ± SD	2.33 ± 1	2.24 ± 0.9	1.7 ± 1

Abbreviation: SD, standard deviation.

No statistically significant differences in range of motion were observed between the operated and the contralateral shoulder at final follow‐up.

### Sports activity and return to sport

No statistically significant differences were observed in the overall sports activity levels between preoperatively and postoperatively (*p* = 0.261) (Table 3).

The median return to sports level was 2 (1–3), equivalent to 90%–100% of previous athletic activity. No difference in return to sports level was observed between patients with recurrence (3 [2.5–3.3]) and those without recurrence (2 [1–3]; *p* = 0.068). The return to sports levels of patients who underwent capsular plication using suture‐only fixation were comparable to those with suture anchor fixation (*p* = 0.104).

## DISCUSSION

Arthroscopic Bankart repair with additional posteroinferior plication in patients with anterior shoulder instability and hyperlaxity without critical bone loss resulted in good clinical outcomes in shoulder function and return to sports at a minimum follow‐up of 10 years, despite a relatively high overall recurrence rate of 18.2%. Suture anchor fixation was associated with a lower recurrence rate (11.1%) compared with suture‐only fixation (50%). A higher number of preoperative dislocations was correlated with worse clinical outcome scores.

Hyperlaxity is a common risk factor for recurrent instability [12]. Several clinical studies have demonstrated its significance in failed arthroscopic Bankart repair [1, 6, 25]. Various surgical techniques have been described for treating anterior shoulder instability in patients with hyperlaxity and no critical bone loss to restore the anatomy and reduce the increased capsular volume [12]. However, the optimal treatment procedure for these patients remains controversial, and long‐term results have not yet been reported.

In our study, arthroscopic Bankart repair was performed using at least three anchors, combined with additional posteroinferior capsular plication using either a suture‐only fixation or a suture anchor fixation. A biomechanical study by Mayer et al. suggests that combining anterior and posterior volume reduction can restore physiological capsular volume and glenohumeral translation from a lax to a native capsular state [21]. The anteroinferior capsular volume reduction was achieved through the capsulolabral reattachment and shift during the Bankart repair, while the posteroinferior capsular volume was reduced by the posteroinferior capsular plication.

Before 2010, the posteroinferior capsular plication was performed using suture‐only fixation as described by Wolf et al. [36], while suture anchor plication was used after 2010. Provencher et al. compared the suture capsulolabral plication to an intact labrum with the glenoid bone anchor fixation in a biomechanical study and found a lower risk of labral displacement when using a suture anchor compared to the suture‐only fixation [24]. They recommended the use of suture anchors, if the labral integrity could be compromised. This could explain the findings in our study, as all patients had a capsulolabral lesion. In our study, the use of the suture‐only fixation was associated with a significantly higher recurrence rate (50%) compared to the use of a suture anchor fixation for the posteroinferior capsular plication (11.1%), suggesting that the use of suture anchors should be preferred in subsequent procedures. Another explanation for the differences in recurrence rates may be the use of different suture materials, as suture‐only plication was performed using absorbable PDS sutures, whereas suture anchor fixation was performed using non‐absorbable sutures. However, these findings should be interpreted with caution given the small sample size and the high loss to follow‐up.

Long‐term results after isolated Bankart repair without posteroinferior capsular plication have shown high recurrence rates between 18% and 37% [22]. A meta‐analysis of risk factors for recurrence after Bankart repair, including 356 patients with a mean follow‐up of 3.4 years, reported a higher recurrence rate in patients with hyperlaxity (28.7%) compared with those without (19.2%). Patients with hyperlaxity were 4.5 times more likely to experience recurrent instability [37]. The lower recurrence rate of 11.1% observed with the additional posteroinferior capsular plication using suture anchor fixation suggests that the addition of the capsular plication may reduce the recurrence rates, even in patients without hyperlaxity. A biomechanical study by Werner et al. demonstrated that, in the presence of an engaging Hill–Sachs defect, combining Bankart repair with posterior capsular plication reduced anterior shoulder instability and Hill–Sachs engagement similarly to Bankart repair with remplissage.

Furthermore, a higher number of preoperative dislocations was significantly correlated with poorer clinical outcomes. Additionally, a correlation between a longer interval between the first dislocation and surgery and poorer clinical outcomes was observed, although these were not statistically significant. Nevertheless, these findings suggest that patients with a high risk of redislocation should be considered for an early surgical treatment to achieve better clinical outcomes.

All recurrences occurred within the first 6 years, which is consistent with the findings of Vermeulen et al., where all recurrences developed within the first 5 years [33]. However, other studies have shown that recurrent instability can occur even more than 10 years after arthroscopic stabilisation [9, 16]. In a study by Hinz et al. with a median follow‐up of 23 years, 12 of 28 patients (42.9%) with recurrent instability experienced recurrence after 10 years [16].

## LIMITATIONS

This study has several limitations due to its retrospective design. Of the 54 initially included shoulders, only 33 (61.1%) were available for follow‐up, resulting in a high lost‐to‐follow‐up rate and a small number of cases. Additionally, no preoperative scores were available for comparison with the postoperative outcomes. The sizes of the two treatment groups were unequal, and follow‐up duration differed significantly between groups, which may have introduced statistical bias and limited statistical power. Suture‐only fixation was used before 2010, whereas the suture anchor fixation was used thereafter. Therefore, the comparison between the two techniques may have been influenced by temporal changes in surgical experience, implants, and patient selection, and the results should be interpreted with caution. However, all recurrences occurred within the first 6 years after surgery, suggesting that the difference in follow‐up periods between the groups likely had no major impact on the results. Different anchor and suture types were used over the long follow‐up period. However, no significant differences in recurrence rates or clinical outcomes were observed between the different anchor and suture types. Furthermore, preoperative images were no longer available for nine shoulders, requiring us to rely on operation reports and intraoperative arthroscopic images to evaluate bone loss. No radiographs were conducted at follow‐up to assess potential osteoarthritis.

## CONCLUSION

Arthroscopic Bankart repair combined with posteroinferior capsular plication provided durable long‐term shoulder function and reliable return to sport in patients with anterior instability and hyperlaxity. Performing the posteroinferior plication with suture anchor fixation might be associated with lower recurrence rates compared to suture‐only plication. Clinical outcomes declined with an increasing number of preoperative dislocations.

## AUTHOR CONTRIBUTIONS

Anh Do was involved in project conceptualisation, collected and analysed the data and prepared the manuscript. Agahan Hayta and Rony‐Orijit Dey Hazra contributed to data collection and revision of the manuscript. Philipp Moroder and Markus Scheibel contributed to project conceptualisation, revised the manuscript and performed the surgeries. Alp Paksoy and Doruk Akgün were involved in project conceptualisation, facilitated data collection, revised the manuscript and supervised the study. All authors have read and approved the final manuscript.

## CONFLICT OF INTEREST STATEMENT

The authors declare no conflicts of interest.

## ETHICS STATEMENT

The study protocol (Application number: EA4/240/23) was reviewed and approved by the institutional review board (Charité – Universitätsmedizin Berlin). All patients provided written informed consent to participate in the study.

## Supporting information

Supporting File

## Data Availability

The data that support the findings of this study are available on request from the corresponding author.
